# Fu’s subcutaneous needling for intractable hiccups: A case report

**DOI:** 10.1097/MD.0000000000043535

**Published:** 2025-08-01

**Authors:** Lu-yao Dou, Hua-feng Cui, Li-Wei Chou

**Affiliations:** a School of Acupuncture-Moxibustion and Tuina, Shandong University of Traditional Chinese Medicine, Jinan, Shandong Province, China; b Department of Acupuncture and Moxibustion, Affiliated Hospital of Shandong University of Traditional Chinese Medicine, Jinan, Shandong Province, China; c Department of Physical Medicine and Rehabilitation, China Medical University Hospital, Taichung, Taiwan; d Department of Physical Therapy and Graduate Institute of Rehabilitation Science, China Medical University, Taichung, Taiwan; e Department of Physical Medicine and Rehabilitation, Asia University Hospital, Asia University, Taichung, Taiwan.

**Keywords:** diaphragm, Fu’s subcutaneous needling, Hiccup Assessment Instrument, intractable hiccups, myofascial trigger points

## Abstract

**Rationale:**

Intractable hiccups pose a significant impact on patients’ quality of life and mental well-being, yet effective nonpharmacological interventions remain limited. Fu’s subcutaneous needling (FSN), a novel acupuncture technique targeting myofascial trigger points (MTrPs), has not been previously reported in treating intractable hiccups.

**Patient concerns:**

The patient developed intractable hiccups (30–40 episodes/min) following treatment for allergic cough, unresponsive to medications and conventional acupuncture.

**Diagnoses:**

Intractable hiccups.

**Interventions:**

FSN was applied to MTrPs in rectus abdominis, psoas major, erector spinae, and adductor magnus muscles. The needle was inserted subcutaneously toward tightened muscles, followed by fan-shaped sweeping for 2 minutes per session.

**Outcomes:**

Hiccup frequency decreased from 6/10 to 1/10 on the Hiccup Assessment Instrument after 7 sessions. Symptoms resolved completely with no recurrence at 1-month follow-up.

**Lessons:**

FSN may be a safe and effective alternative for intractable hiccups by inactivating MTrPs and modulating fascial tension. Further randomized controlled trials are warranted.

## 1. Introduction

Hiccups are triggered by the abrupt, involuntary contraction of the diaphragm and intercostal muscles, leading to the momentary closure of the glottis. When hiccups persist for over a month, they are classified as intractable hiccups.^[1]^ Several medications have proven effective; however, drug therapy has various adverse reactions.^[2]^ Studies have reported that chlorpromazine may induce the occurrence of intrahepatic cholestasis and agranulocytosis,^[3,4]^ while baclofen can cause drowsiness and fatigue.^[5]^ Physical therapies such as acupuncture, peripheral nerve stimulation, vagus nerve stimulation, and peppermint inhalation have also demonstrated efficacy in treating hiccups^[6–9]^, but their therapeutic effects are uncertain.^[10,11]^ The clinical management of intractable hiccups remains challenging. Therefore, it is necessary to search for a treatment method with minor side effects and stable therapeutic effects.

This case report presents an innovative case of intractable hiccups successfully treated with Fu’s subcutaneous needling (FSN), aiming to contribute novel insights into treating this condition. The patient was admitted to the hospital due to allergic cough and developed intractable hiccups after a series of treatments, and conventional treatments failed to provide relief, significantly impacting his quality of life.

FSN has shown promising results in pain management, particularly in rapidly alleviating pain associated with musculoskeletal disorders.^[12–14]^ And it also has a good therapeutic effect on other nonmusculoskeletal medication problems.^[15–17]^ However, its application in treating intractable hiccups has not been previously reported. Therefore, we present this case to illustrate the feasibility and potential of FSN in addressing intractable hiccups.

## 2. Case presentation

Adolescent male, visited in September 2024. The patient visited a local hospital on August 1 due to persistent allergic cough. He had a 10-year history of allergic cough and had been treated with “inhaled glucocorticoids and long-acting inhaled β-adrenergic receptor agonists” for a long time. He began to receive regular subcutaneous injections of omalizumab every month from April 2024 and completed the fifth injection on August 1, 2024. He had a history of liver cysts, gallstones, gastritis, and ethmoid sinusitis. He had an allergic constitution since childhood and a history of allergies to cephalosporins and penicillins. He reported that he had chest pain and dizziness after receiving the COVID-19 vaccine in 2021. Gastrointestinal Barium Meal in April 2024: Findings are consistent with gastritis. Immunoglobulin IgE quantitation on August 9, 2024: 660.0 IU/mL, erythrocyte sedimentation rate: 20.00 mm/h. Treatment was administered with budesonide, glycopyrronium and formoterol inhalation aerosol, intravenous infusion of omeprazole, and subcutaneous injection of Omalizumab.

The patient developed intractable hiccups on August 3. The hiccups were characterized by persistent episodes, each lasting over 30 minutes, with only brief remissions of 1 to 2 minutes before recurrence. Symptoms occurred continuously both day and night and were exacerbated by fatigue. The hiccups were short and strong, with a frequency of about 30 to 40 times per minute. This condition resulted in severe sleep disturbance (nocturnal sleep limited to 3–4 hours), difficulty eating, and low mood, ultimately leading to temporary leave from school.

The patient sought initial treatment at a local hospital, where intramuscular metoclopramide and oral omeprazole were administered. However, no significant symptomatic relief was achieved, and hiccups persisted. On August 18, the patient was referred to the acupuncture department of our hospital. Adjunctive therapies including acupuncture, tuina massage, and acupotomy were initiated, yet hiccup episodes remained unabated. Therefore, the patient presented to our outpatient clinic specifically seeking FSN therapy in September. The Hiccup Assessment Instrument (HAI),^[18]^ a subjective measure of hiccup severity, initially rated the patient’s preprocedural score as “moderate” with a score of 6/10 before FSN treatment.

**Figure 1.**
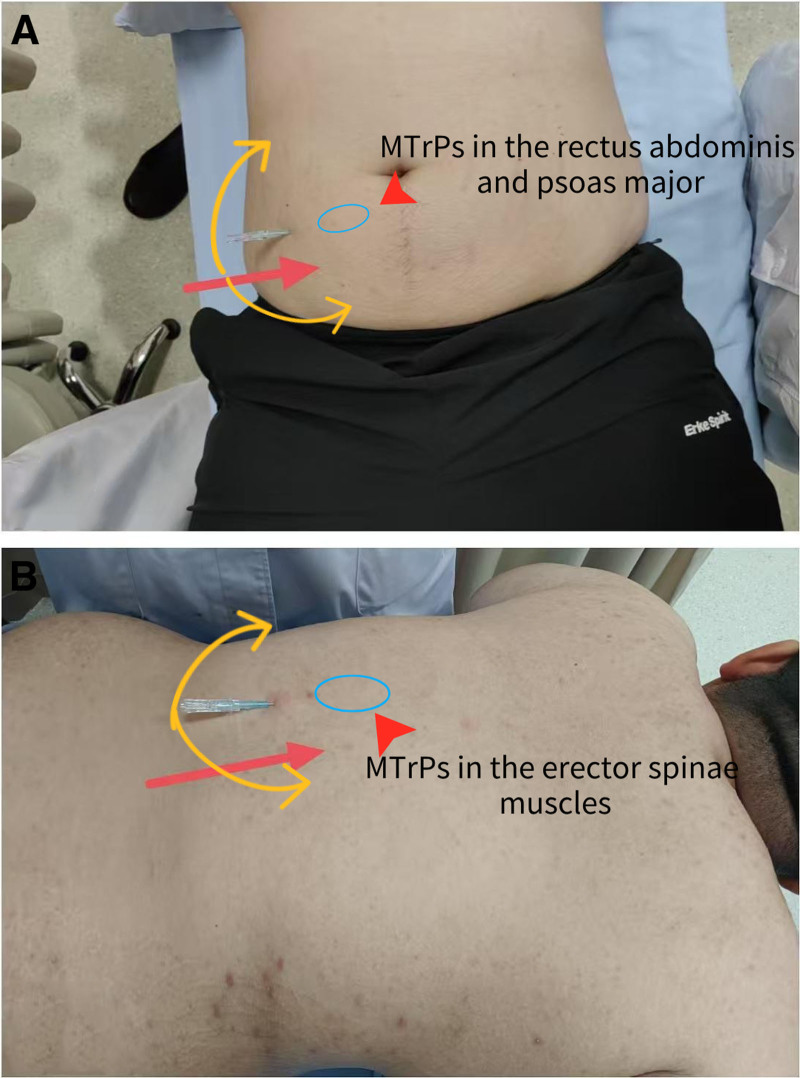
(A) The rectus abdominis muscles and psoas major are the affected muscle, and needling direction is indicated by red arrows, with swaying at 40° in a fan-shaped pattern indicated by yellow arrows. (B) The erector spinae muscle is the affected muscle, and needling direction is indicated by red arrows, with swaying at 40° in a fan-shaped pattern indicated by yellow arrows. MTrPs = myofascial trigger points.

**Figure 2.**
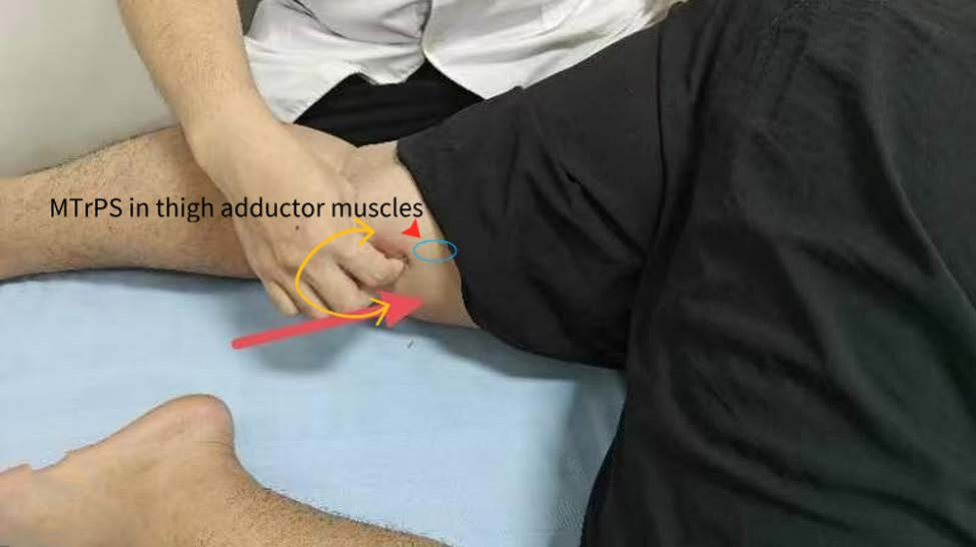
The thigh adductor muscles are the affected muscle, and needling direction is indicated by red arrows, with swaying at 40° in a fan-shaped pattern indicated by yellow arrows. MTrPs = myofascial trigger points.

Through palpation, myofascial trigger points (MTrPs) were found in the patient’s rectus abdominis, erector spinae, psoas major, and thigh adductor muscles. FSN mainly treats the disease by addressing these MTrPs, promoting blood reperfusion to the affected area, and reducing muscle stiffness.^[19]^ The physician selected the muscle that has 1 or more MTrPs, and feels cold, stiffness, numbness, or painful to the patient as the affected muscle, so called “tightened muscle (TM),”^[20]^ including rectus abdominis muscles, psoas major, erector spinae muscles (Fig. 1), and thigh adductor muscles (Fig. 2). The “TM” is the treatment target. Three needle insertion points were selected for each treatment session:

1. Abdominis muscles and psoas major: the junction of the middle and outer thirds of the line connecting the umbilicus to the right anterior superior iliac spine;
2. Erector spinae muscles: Over the left erector spinae muscle in its mid-lower segment, approximately at the level of the T10 vertebra;
3. Thigh adductor muscles: Approximately 3 cm above the left medial femoral condyle.

**Figure 3.**
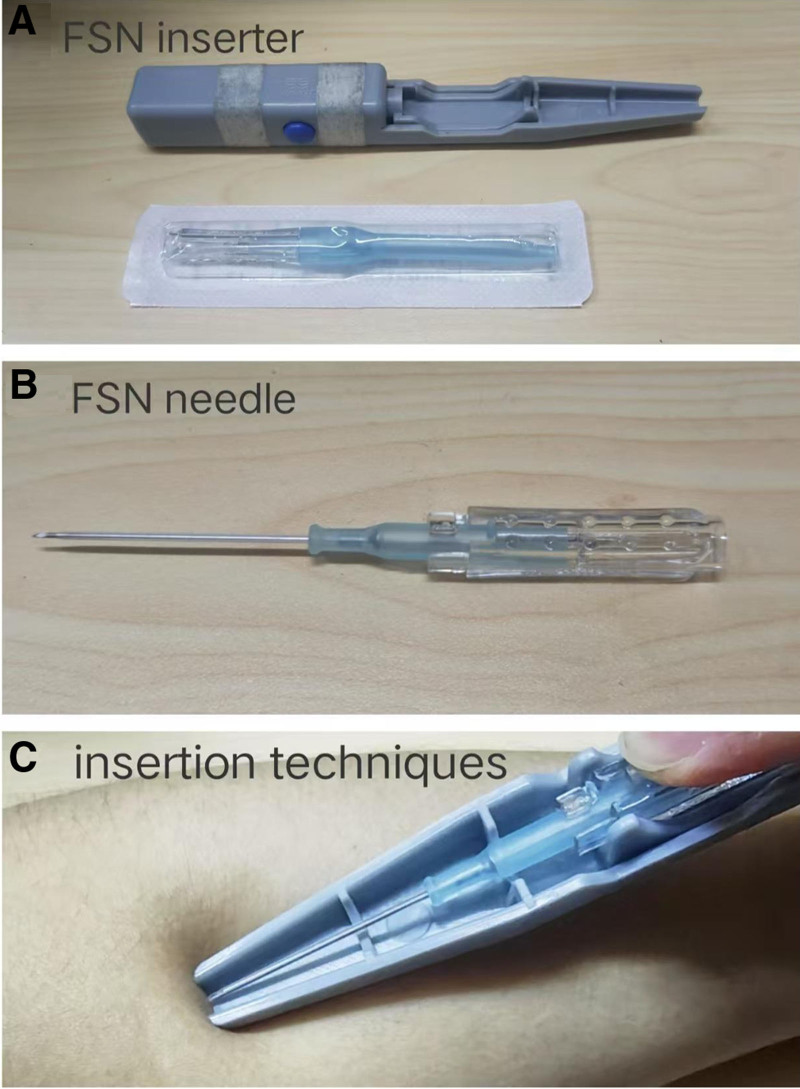
(A) FSN inserter. (B) FSN needle. (C) Insertion techniques of FSN. FSN = Fu’s subcutaneous needling.

First, routine disinfection of the needle insertion point is performed. Using the thumb and index finger of the left hand, the skin is fixed. Holding the FSN inserter in the right hand at an angle of 15° to 20° to the skin, propel the medium-gauge disposable FSN (Nanjing Paifu Medical Technology Co., Ltd.; Batch No. 20152270832) parallelly into the loose subcutaneous connective tissue at the insertion point(Fig. 3), advancing it toward the “TM.” The needle body is then slowly advanced along the superficial fascia layer, until the needle body is fully inserted subcutaneously, ensuring no pain, soreness, numbness, or swelling is experienced during insertion. Once the needle is properly positioned, the needle tip is withdrawn into the soft tube sleeve. The thumb and middle finger of the right hand are used as a fulcrum, while the index finger and ring finger are placed on both sides of the needle body. Fan-shaped sweeping movements are performed at an angle of 40° with a frequency of 100 times per minute for 2 minutes. Each treatment session lasts for a total duration of 10 minutes. After completing these actions, the needle core is removed, leaving the soft tube under the skin of the thigh’s adductor muscles, which is then fixed with medical tape. The soft cannula remains in place for 5 hours before being removed by the patient. No additional medications are administered during the treatment.

**Figure 4.**
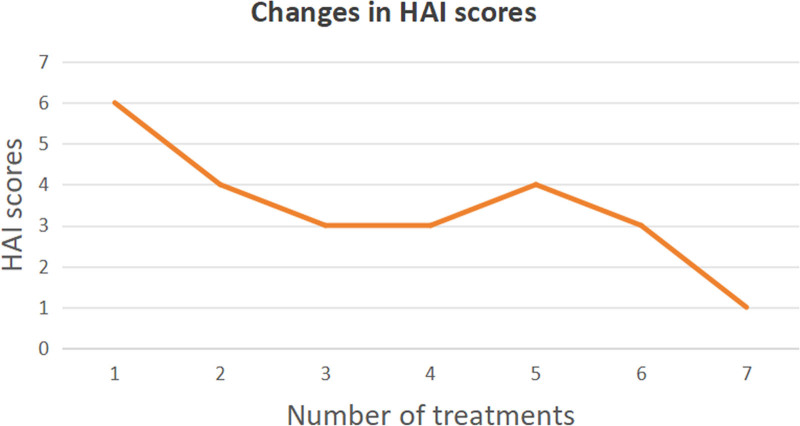
Changes in HAI scores. HAI = Hiccup Assessment Instrument.

Following one treatment, the patient reported relief from hiccups, with an HAI score of “4/moderate.” The patient underwent daily treatments for a total of 7 sessions. After the final treatment on September 10, 2024, hiccup symptoms were nearly absent, and the HAI score improved to “1/mild” (Fig. 4). The patient’s hiccup symptoms completely resolved on September 12, 2024. On September 13, 2024, the local hospital continued the omalizumab injection according to the prescribed course of treatment. During the treatment, the patient reported no discomfort and exhibited no adverse reactions. The patient described the needling process as causing only mild, acceptable pain. At the 1-month posttreatment telephone follow-up, the patient reported complete resolution of hiccups without recurrence.

## 3. Discussion

The precise cause of hiccups remains incompletely understood. The widely accepted theory is the “hiccup reflex arc.”^[21]^ When certain stimuli are encountered, signals are transmitted to the central nervous system via the vagus nerve, phrenic nerve, and sympathetic nerve fibers (originating from spinal segments T6 to T12). The phrenic nerve functions as the reflex efferent pathway in this process. During the reflex, the pertinent nerve centers are situated in the spinal cord region from C3 to C5, in proximity to the respiratory center of the medulla oblongata, and also implicate the reticular formation and hypothalamus of the brainstem. Excessive stimulation of any component of this reflex pathway may elicit hiccups.^[7]^ Common triggers of hiccups include overeating, rapid eating, and bloating due to the consumption of carbonated beverages. For intractable hiccups, it is essential to conduct necessary examinations to exclude underlying organic conditions such as cerebrovascular disease, tumors, and reflux esophagitis. In many patients, stimulation of the vagus nerve and phrenic nerve is a frequent cause of intractable hiccups.^[22]^ The diaphragm serves as the main respiratory muscle. A study^[23]^ suggests that chronic airflow limitation can alter the position of the diaphragm, subsequently impacting inspiratory muscle function. The persistent hiccups reported in this case might be attributed to the patient’s continuous coughing, which caused abdominal muscle tension and diaphragmatic spasms, resulting in the hiccups.

Acupuncture has been demonstrated to be an effective treatment for hiccups.^[24]^ FSN, a novel acupuncture technique invented by Dr Fu Zhonghua in 1996, primarily targets the superficial fascia layer, specifically the loose connective tissue beneath the skin. By employing a swaying movement, this technique elicits piezoelectric and reverse piezoelectric effects in the subcutaneous loose connective tissue, converting electrical currents into the necessary mechanical or chemical energy to restore normal muscle physiological function.^[25]^ A study^[15]^ reported that Fu’s subcutaneous needle therapy can improve dyspnea symptoms in patients with severe diaphragmatic dysfunction and reduce the duration of noninvasive ventilation. Fu’s subcutaneous needle therapy can inactivate myofascial trigger points in muscles, reduce muscle spasms, and promote gastrointestinal motility.^[16]^ Studies have shown that the rectus abdominis muscles and lumbar and back muscles are connected to the diaphragm through fascia. When the myofascial trigger points in the abdominal and back muscles lose their activity, the MTrPs in the related diaphragm may also disappear.^[26]^ The deep anterior line functions as the pivotal myofascial structure, stabilizing the thorax during respiration. It spans from the foot sole, ascending along the posterior calf, through the adductor muscles and the psoas major, and continuing upwards along the posterior abdominal wall to connect with the diaphragm crus and posterior diaphragm, ultimately reaching the head and face. Notably, the psoas major is vital for stabilizing the thoracic-lumbar spine-pelvis-hip joint complex. Modern anatomical insights reveal fascia as a pervasive and continuous network throughout the body, giving rise to the concept of the myofascial chain.^[27]^ Research indicates that treatment based on the fascial chain’s remote effects is comparable to local treatment in efficacy. Myofascial tissue has the ability to transmit tension to other muscles.^[28–30]^ Given these insights, this case report selected specific points for needle insertion: the rectus abdominis muscles, thigh adductor muscles, and psoas major along the deep anterior line. These muscles not only constitute the core structure of the deep anterior line but are also connected to other parts of the body through fascial chains, forming a continuous mechanical transmission network. FSN at these points can stimulate the tension transmission of the fascial chains, thereby affecting the distant diaphragm. Additionally, the middle segment of the erector spinae muscle at the tenth thoracic vertebra was targeted for local fascial sweeping. This approach aims to suppress the overexcitement of the vagus nerve, thereby effectively preventing the occurrence of hiccups.

The mechanism of FSN for intractable hiccups remains unclear, but it may be related to the elimination of myofascial trigger points in tense muscles. Myofascial trigger points are the most tender (highly irritable) points found within taut bands in skeletal muscle fibers, and can cause skeletal muscle pain.^[31]^ Excessive release of acetylcholine in abnormal endplates may generate taut bands in skeletal muscle fibers that contain myofascial trigger points.^[32]^ Studies^[19,33]^ have shown that FSN can effectively reduce the number of myofascial trigger points and improve pain.

To our knowledge, this is the first reported case of using FSN to treat intractable hiccups induced by a series of treatments for allergic cough. There is no unified treatment protocol for intractable hiccups, and previous studies have primarily focused on the vagus nerve. This case report innovatively addresses the issue from the perspective of muscles, aiming to relieve hiccups by eliminating myofascial trigger points and alleviating muscle tension. FSN boasts advantages of safety and efficiency. It targets the superficial fascia beneath the skin and employs a flat puncture insertion method, rendering the procedure virtually painless and noninvasive. This therapy is devoid of complications and is readily acceptable to all.

## 4. Conclusion

Intractable hiccups significantly impair patients’ quality of life, physical health, and psychological well-being, with frequent comorbidity of anxiety and depression due to persistent symptoms. Clinical practice lacks standardized treatment plans for this condition. When conventional pharmacological interventions fail due to limited efficacy or adverse effects, FSN, a novel acupuncture-derived technique, may serve as a safe, noninvasive, and potentially effective complementary therapy. Our case demonstrated rapid symptom resolution (HAI score reduced from 6/10 to 1/10) with no recurrence during follow-up, suggesting its clinical feasibility. However, this report pertains to a single case and lacks robust clinical evidence. Future research should involve multicenter, large-sample randomized controlled trials for validation. Additionally, while FSN’s therapeutic mechanisms may involve myofascial chain-mediated modulation of diaphragmatic tension and vagal nerve activity, direct evidence linking mechanical stimulation to neurophysiological changes is lacking. Further investigations combining biomechanical modeling and functional imaging are warranted to elucidate these pathways.

## Author contributions

**Investigation:** Lu-yao Dou.

**Writing – original draft:** Lu-yao Dou.

**Resources:** Hua-feng Cui, Li-Wei Chou.

**Supervision:** Hua-feng Cui, Li-Wei Chou.

**Writing – review & editing:** Hua-feng Cui, Li-Wei Chou.
